# Genomic Multicopy Loci Targeted by Current Forensic Quantitative PCR Assays

**DOI:** 10.3390/genes15101299

**Published:** 2024-10-05

**Authors:** Richard Jäger

**Affiliations:** 1 Department of Natural Sciences, Bonn-Rhein-Sieg University of Applied Sciences, von-Liebig Str. 20, 53359 Rheinbach, Germany; richard.jaeger@h-brs.de; 2 Institute for Functional Gene Analytics, Bonn-Rhein-Sieg University of Applied Sciences, Grantham Allee 20, 53757 Sankt Augustin, Germany; 3 Institute of Safety and Security Research, Hochschule Bonn-Rhein-Sieg, University of Applied Sciences, Grantham Allee 20, 53757 Sankt Augustin, Germany

**Keywords:** quantitative PCR, qPCR, real-time PCR, forensic, STR, Alu, Y chromosome, DNA degradation, Plexor, TaqMan

## Abstract

Modern forensic DNA quantitation assays provide information on the suitability of a DNA extract for a particular type of analysis, on the amount of sample to put into the analysis in order to yield an optimal (or best possible) result, and on the requirement for optional steps to improve the analysis. To achieve a high sensitivity and specificity, these assays are based on quantitative PCR (qPCR) and analyze target DNA loci that are present in multiple copies distributed across the genome. These target loci allow the determination of the amount of DNA, the degree of DNA degradation, and the proportion of DNA from male contributors. In addition, internal control DNA of a known amount is analyzed in order to inform about the presence of PCR inhibitors. These assays are nowadays provided as commercial kits that have been technically validated and are compatible with common qPCR instruments. In this review, the principles of forensic qPCR assays will be explained, followed by information on the nature of DNA loci targeted by modern forensic qPCR assays. Finally, we critically draw attention to the current trend of manufacturers not to disclose the exact nature of the target loci of their commercial kits.

## 1. Introduction

Forensic short tandem repeat (STR) analysis requires optimal DNA amounts. Too low amounts will fall below the analytical sensitivity and entail stochastic effects, resulting in a loss of information; too high amounts will cause analytical artefacts. To adjust the optimal DNA amounts for the subsequent analysis and to identify promising or unsuitable samples, it is important to quantitate the DNA that has been extracted from trace material. In human forensics, the quantitation methods should specifically measure human (and not contaminating microbial, animal- or plant-derived) DNA. Furthermore they should be highly sensitive such that only a small proportion of a precious sample is consumed for the quantitation. For these reasons, nowadays, quantitative PCR (qPCR)-based methods are used. These offer the further advantage of being able to simultaneously analyze several target DNA loci in a multiplex analysis. By this means they can provide additional information, such as on DNA degradation and the presence of PCR inhibitors or male components, while at the same time consuming less sample and reducing the time of the analysis. In this review, the technical principles of qPCR methods currently used in forensics are briefly explained, followed by considerations on the rationales of the multiplex assay design. Finally, the DNA loci targeted by current qPCR assays are discussed.

## 2. Principles of qPCR

Quantitative PCR is a relative quantitation method based on comparing measurements of an unknown sample with measurements of standard samples of known DNA concentrations to calculate the concentration of the unknown. The principle consists in monitoring the accumulation of PCR products in real time and determining the number of PCR cycles required to reach a certain threshold (called the threshold cycle, C_t_, or quantification cycle, C_q_), typically set in the exponential phase of the PCR [1]. The higher the template DNA amount, the fewer PCR cycles are required to reach the amplification threshold and thus the lower the C_t_ value will be. In its simplest form, product accumulation is measured after the elongation step of each PCR cycle with the help of a DNA-binding fluorescent dye present in the reaction mix, such as SYBR green [2]. As such dyes cannot distinguish between different PCR products, SYBR green-based qPCR cannot be multiplexed, and the specificity has to be confirmed by a further method (such as melting curve analysis [3]). For these reasons, forensic qPCR assays are not based on DNA-binding fluorescent dyes.

**Figure 1:**
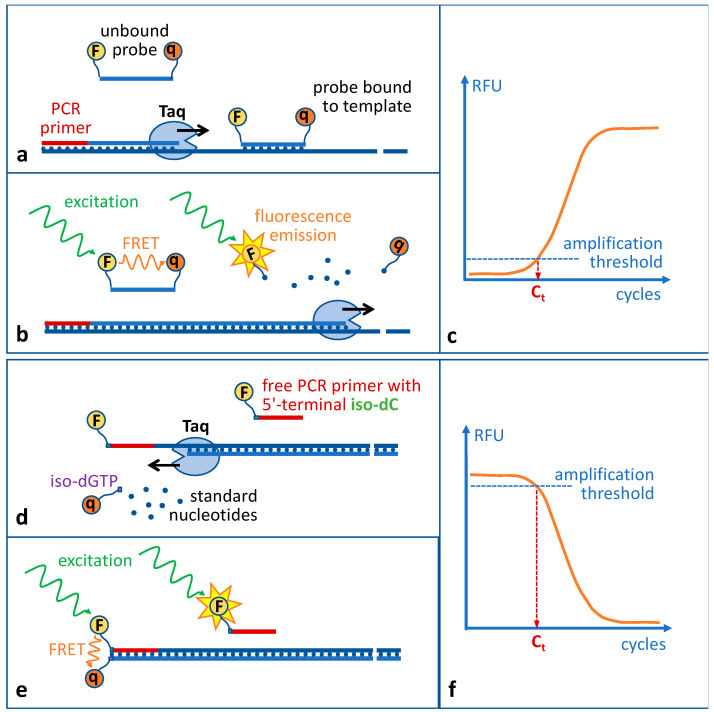
Detection of PCR products in qPCR using TaqMan probes (**a**–**c**) or Plexor technology (**d**–**f**). (**a**) A fraction of the TaqMan probes (probe) is annealed to the template strand after the annealing step of PCR. (**b**) During elongation, the template-bound probe is degraded due to the 5′–3′ exonuclease activity of the Taq polymerase, releasing the fluorophore (F) from the quencher (q). Excitation at the end of the elongation step thus leads to fluorescence emission by the released fluorophore, while the fluorescence emission of the unbound probe is quenched by Förster resonance energy transfer (FRET). (**c**) Thus, fluorescence emission (expressed in RFU, relative fluorescence units) is proportional to the number of elongated template copies and increases with each cycle. The C_t_ value is the cycle number required to reach a certain amplification threshold, which is typically set within the exponential amplification phase. (**d**) In the Plexor technology, one of the two PCR primers carries an unusual base (iso-dC in this example) at its 5′ end that is coupled to a fluorophore (F). Thanks to the primer, previously elongated template molecules carry the iso-dC at their 5′ end. (**e**) Elongation in the presence of a quencher-linked unusual nucleotide (iso-dGTP) specifically base pairing with the iso-dC leads to incorporation of the iso-dG and quenching of the fluorescence emission by FRET, whereas the free primer emits light upon excitation. (**f**) Therefore, the number of light-emitting free primers decreases with each PCR cycle, and the C_t_ value is determined with the help of an amplification threshold set through the exponential decrease in fluorescence.

In most forensic qPCR assays, the detection of PCR products is accomplished using dual-labeled hydrolysis probes (also called TaqMan probes) that moreover confer an additional level of target specificity [1,4]. These are short oligonucleotides present in the PCR reaction that bind to one strand of the target amplicon between the primer binding sequences (see Figure 1a). On one end, the probes are covalently linked to a fluorophore, the fluorescence emission of which, however, is quenched by a second fluorophore (called quencher) that is attached at the other end of the probe. By its 5′–3′ exonuclease activity, the passing Taq polymerase degrades the probe, thus releasing the quencher from the fluorophore. The light emission by the flurophore is then monitored at the end of the elongation step.

A further occasionally used method (called Plexor technology) is based on the specific base pairing between nucleotides not normally present in DNA [5]. Here, one of the PCR primers contains a fluorophore-labeled unusual base at its 5′ end that will not base pair with standard nucleotides (see Figure 1b). In addition to the four standard nucleotides, the PCR mix contains a second non-standard nucleotide that is covalently linked to a quencher and is specifically base pairing with the unusual nucleotide at the primer-derived 5′ ends of the amplified templates. By incorporating this second nucleotide at the 3′ end of the newly synthesized strand, the signal from the fluorophore at the 5′ end of the template is quenched, allowing for monitoring the PCR product accumulation based on the decreasing fluorescence signal after each elongation step.

## 3. Design of Forensic qPCR Assays

Modern forensic DNA quantitation assays are designed as multiplex qPCR assays that provide quantitative information on several important parameters, such as DNA amount, DNA integrity, the presence of PCR inhibitors, and the male component in DNA mixtures. Specific amplicons for these parameters are amplified in parallel and detected in separate color channels. In addition, some assays contain a passive reference dye to control the amount of reaction mix of each sample. Apart from saving time and sample amount, the multiplex design has the advantage that all parameters are assessed from the same sample fraction, thus increasing accuracy. In the following sections, the principles of the analysis of the different parameters are explained.

### 3.1. Sensitivity and Specificity

**Figure 2:**
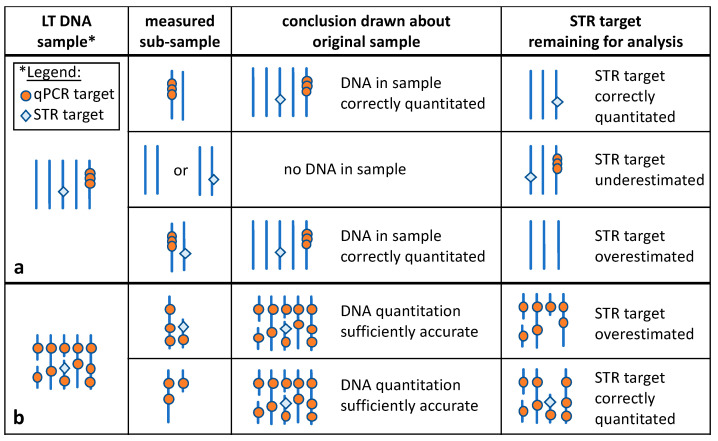
Schematic of possible scenarios for quantitation of a low template (LT) DNA sample containing only one to two genome copies using (**a**) a multicopy target residing on one chromosomal locus (**b**) or a multicopy locus uniformly distributed across the genome. Depending on the composition of the subsample taken for quantitation, different conclusions may be drawn about the DNA amount of the original sample or about the STR target present in the remaining sample. Schematically, only five chromosomes (vertical blue lines) are depicted, of which two chromosomes are present in the sample taken for quantitation in (**a**,**b**). In (**b**), two of the chromosomes are fragmented, and the subsample contains one chromosome and one chromosomal fragment. In cases where a copy of an STR target is removed with the measured subsample, this copy is absent in the sample remaining for analysis, and hence, the copy number is lower than expected from the quantitation result (“STR target overestimated”). Please note that for higher copy numbers, the subsample is likely to represent the original sample, and the number of STR targets (or their proportion) in the remaining sample will not be noticeably affected. Circles: targets for qPCR-based quantitation; diamonds: STR locus to be analyzed.

Modern forensic DNA analysis is able to establish complete STR profiles from as little as 125 pg genomic DNA, corresponding to the DNA of nineteen diploid cells (or 19 diploid genome copies, each 6.6 pg nuclear DNA) [6,7,8]. For even lower DNA amounts, modified protocols have been developed, and thus, it is possible to obtain information from just four to five cells (or genome copies) or even fewer [9,10,11]. Correspondingly, qPCR methods need to be able to reliably quantitate in the picogram range. While PCR is in principle able to amplify single DNA molecules, if single-copy loci or (multicopy targets from a single DNA locus, as shown in Figure 2a) were chosen for quantitation, any such analysis of low template DNA would be at risk of stochastic sampling errors [12], thus possibly underestimating the true DNA amount (see Figure 2a for explanation). To be able to quantitate in the picogram range and to minimize stochastic sampling errors, current forensic qPCR assays analyze genomic loci that are present in many copies per genome and are uniformly distributed across several chromosomes. Thus, irrespective of the genome fraction ending up in the subsample taken for quantitation, the quantitation reflects the overall amount of DNA in the original sample (see Figure 2b). The human genome contains many such multicopy loci that sufficiently differ in sequence from genomic loci of non-human species to allow for the design of human-specific and highly sensitive DNA quantitation assays [13]. Species cross reactivity has yet to be empirically tested when establishing and validating forensic qPCR assays.

Current forensic qPCR assays can accurately quantitate DNA of just 2.5–5 pg/µL [14], and their sensitivity, expressed as the limit of detection (LOD), is even below 1 pg/µL for the latest assays [15,16,17]. Of note, DNA concentrations less than 1 pg/µL are indicative of only a few genome copies present in the sample, resulting in incomplete STR profiles due to stochastic sampling effects [12]. However, at low DNA concentrations, the accuracy of the qPCR assays is low; hence, the measurement may underestimate the true amount of DNA in the sample that may still yield a complete STR profile [18].

Moreover, DNA samples with quantitation results of less than 1 pg/µL bear the stochastic risk that the subsample taken for quantitation has removed copies of some of the STR loci to be analyzed, thus augmenting allelic imbalances or causing allele or locus drop-outs in the subsequent STR analysis (see Figure 2b). Thus, for singular traces that are expected to yield very low amounts of DNA (such as touch DNA or single hair shafts [19]), it may be advisable to dispense with the quantitation in order to increase the chance of a successful STR analysis when applying low copy DNA methods [9,20].

### 3.2. DNA Integrity

The term DNA integrity expresses the intactness of genomic DNA as required for a type of downstream analysis. In forensic STR analysis, information on DNA integrity can thus serve as a predictor of DNA typing success [21,22] and can help identifying samples where alternative DNA markers or DNA typing methods might be required [23,24]. DNA integrity is inversely related to DNA degradation, which is often discussed in terms of single or double strand breaks, caused by microbial or tissue-derived DNases, affecting the STR amplicons. However, environmentally caused chemical damage of the DNA, such as the oxidation, crosslinking, or hydrolysis of bases, will likewise affect STR analysis by impairing primer binding or strand elongation and is as well part of DNA degradation [25].

**Figure 3:**
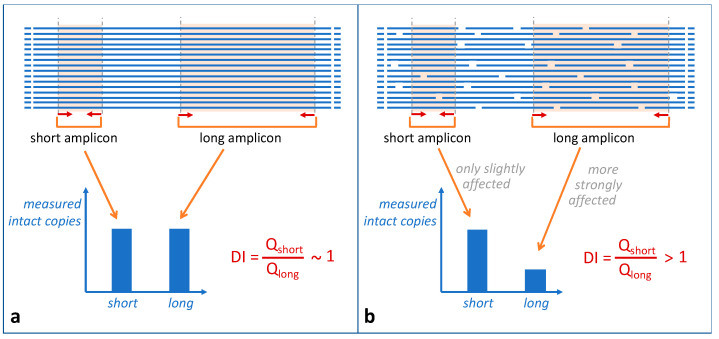
The principle of quantitating DNA degradation by targeting a short and a long amplicon. (**a**) Genomic DNA not degraded and (**b**) degraded genomic DNA. The template DNA copies are depicted as horizontal lines, and the area covered by the two amplicons is highlighted. In (**b**), DNA degradation is indicated by irregular gaps in the template DNA copies. As depicted in (**b**), DNA degradation is more likely to affect the long amplicon, thus reducing the amount of measured intact copies. The degradation index (DI), defined by the ratio of quantitated copies of the short amplicon (Q_short_) and of the long amplicon (Q_long_), will be greater than 1 in the case of DNA degradation.

DNA strand breaks or chemical damage is more likely to occur the longer an amplicon is. The idea to assess DNA integrity by qPCR consists in the quantitation of two amplicons of different lengths, one longer amplicon sensitive to degradation (called the degradation target) and a short one relatively unaffected by degradation (called the quantitation target) [26]. As only intact DNA will be PCR-amplified, the ratio between the two quantitation results correlates with the DNA degradation and is expressed as degradation index (DI) (see Figure 3). The long amplicon of such a qPCR assay typically has a length in the range of the longer STR amplicons of the STR assays, and the short amplicon has a length below the smallest STR amplicon to be analyzed. The typical size range of STR amplicons is from about 100 to 350 bp. Thus, if the degradation target is affected, the longer STR amplicons will be affected as well, resulting in a loss of information.

In first qPCR assays quantitating DNA degradation, two single-copy loci of different amplicon lengths were analyzed [26]. Nowadays, to increase the analytical sensitivity of such assays and to avoid stochastic effects, the quantitation of two differently sized amplicons of multicopy loci is used [7,15,16,27]. Due to their uniform distribution across several chromosomal locations, it is implicitly assumed that their degradation reflects the degradation also of the STR loci of interest and can thus be used to predict STR typing success. The two amplicons should not overlap in order to avoid unpredictable amplification effects due to interference, competition, or amplification of the shorter amplicon from the longer PCR product. This can be achieved by designing non-overlapping amplicons of the same locus or by using two different multicopy loci with roughly similar copy numbers.

### 3.3. PCR Inhibition

Depending on the DNA extraction protocol and the source of DNA, forensic DNA samples may contain impurities that impair PCR by various molecular mechanisms [28]. It is useful to know about the presence of such so-called PCR inhibitors as their presence may mislead the qPCR-based quantitation and likely impairs STR typing as well, such that the sample would not be taken further to STR analysis, or additional measures might be envisaged to overcome the inhibition [28]. PCR inhibition affects longer amplicons more strongly than the shorter ones [29]; thus, the presence of inhibitors may more strongly affect the degradation target (see Section 3.2) and misleadingly suggest DNA degradation. Attempts to deal with degradation by increasing the sample amount will thus introduce even more inhibitor and impair the STR analysis even further.

To detect PCR inhibition, modern forensic qPCR assays contain a so-called internal PCR control (IPC) that is a synthetic template DNA of a known amount that is efficiently PCR-amplified by a dedicated primer pair present in the reaction mix. The amplification of the IPC is sensitive to PCR inhibitors; thus, a shift to higher C_t_ values than to be expected for the IPC input is indicative of PCR inhibitors present in the sample. Modern commercial STR assays are rendered robust against PCR inhibitors and can deal with PCR-inhibiting impurities up to a certain concentration [30]. Thus, the amplification of the IPC should be sensitive to PCR inhibitor concentrations above those tolerated by STR assays. A comparison of current qPCR assays has revealed that while they all were equally able to detect the presence of PCR inhibitors, for some assays the DNA quantitation results were affected by higher inhibitor concentrations [14].

### 3.4. Male Contributors

In sexual assault cases, intimate swabs are analyzed that typically contain a mixture of victim DNA and perpetrator-derived DNA [31]. In the majority of cases, the victims are female and the perpetrators are male. To see whether a DNA analysis might have a chance to reveal the male perpetrator by typing autosomal STRs or Y-chromosomal STRs (Y-STRs), it is useful to quantitate the proportion of male-derived DNA in the total extract [32,33]. Moreover, for some forensic questions, such as on archeological or historic samples, Y-STR typing may yield useful information, e.g., on genealogy or family relations [34]. To these ends, modern qPCR multiplexes target Y-chromosomal sequences, typically from Y-specific multicopy loci to increase the analytical sensitivity.

## 4. Multicopy Loci Used in Forensic qPCR Assays

Modern forensic qPCR assays analyze mostly multicopy loci that are present on the autosomes (and on the gonosomes) or specifically on the Y chromosome and by this means comply with the high sensitivity of modern STR kits. Recently, the diversity and copy number of multicopy DNA loci in various human populations has comprehensively been analyzed using data from the 1000 Genomes Project [13]. Ideally, loci used for forensic qPCR assays should be present in dozens to thousands of copies, should be uniformly distributed across the genome (or the Y chromosome), should display little variation in copy number inter-individually or across populations, should show little sequence variation, should be human-specific, and should be robustly and specifically PCR-amplifiable. This section gives information on the multicopy target loci of current forensic qPCR assays. An overview is given in Table 1 that also shows that for the latest forensic qPCR kits on the market, the identities, chromosomal localizations, and copy numbers of the target loci have not been disclosed. The tendency to keep information on target loci confidential will be critically discussed at the end of this section.

### 4.1. Transposable Elements

Ideal candidates for qPCR-suited multicopy genes are retrotransposons, some of which are dispersed in high copy numbers throughout the genome and emerged only during primate or human evolution and thus are primate- or even human-specific [38]. Retrotransposons are a subclass of transposons, genetic elements that are integrated in the genome and can, by enzymatically catalyzed mechanisms, “jump” to other chromosomal locations. Retrotransposons do so in a copy-paste fashion by being transcribed into an RNA that integrates by reverse transcription-based mechanisms at other places in the genome (reviewed in [38]). They can be classified as autonomous retrotransposons (LINEs, long interspersed elements) and non-autonomous retrotransposons to which short interspersed elements (SINEs) and SINE-VNTR-Alu elements (SVAs) belong. Various mechanisms evolved in organisms that counteract transposition. Yet, over time, by means of the copy-paste jumping mechanism, some retrotransposons have populated large parts of the genomes, thus contributing to evolution but in individuals also adversely affecting gene function and causing disease [39].

L1 is the major LINE in primates. With a length of 6 kb and over 500,000 copies, L1 sequences make up roughly 17% of the human genome [38]. LINEs encode the enzymatic machinery (e.g., reverse transcriptase) required for transposition. Non-autonomous retrotransposons do not encode these enzymes and jump with the help of the enzymes expressed by LINEs. The predominant SINEs in primates are the *Alu* elements (reviewed in [38]). *Alu* elements are non-coding and have sizes of about 300 bp and terminal poly(dA) tails with varying lengths that lead to length variations of the *Alu* elements [40]. With about one million copies they make up roughly 10% of the human genome. *Alu* elements were acquired late during primate evolution and are thus specific for higher non-human primates and humans. *Alu* elements were among the first multicopy loci used in highly sensitive, human-specific forensic qPCR assays [41]. There are three major *Alu* subfamilies that evolved in primates (*Alu*J, *Alu*S, and *Alu*Y—evolutionary age in this order) and can be further subdivided into lineages. Of these, the *Alu*Yb lineage is the second largest young *Alu*Y group and has 1733 copies in the human genome that are distributed across all chromosomes [42]. The *AluYb8* is used in the commercial Innoquant HY kit, which also uses an SVA as the target locus (see Table 1 and [16]).

The SVAs consist of *Alu*-like sequences, followed by a variable number tandem repeat (VNTR) and a SINE-R-like sequence; they are the evolutionarily youngest group of retrotransposons and have only a few thousand copies per human genome, each with a size of about 2 kb [43]. Differences in VNTR repeat numbers are responsible for inter-individual length variations. SVAs evolved in primates and are composed of six subfamilies, two of which are even human-specific [43].

### 4.2. Autosomal Multicopy Loci That Are Not Retrotransposons

Multicopy loci other than retrotransposons have been established for forensic qPCR assays as well. Candidates for genetic loci that are present in high copy numbers are genes that encode non-translated RNAs, some of which are in high cellular demand to sustain cellular functioning. For the majority of these RNA genes the functional significance of their high copy numbers is not clear [44].

One class of candidates for multicopy loci are genes for small nuclear RNAs (snRNAs), which are components of the spliceosomes involved in pre-mRNA splicing [45]. The *RNU2* locus is used in the commercial Plexor HY kit (see Table 1 and [27]) and in the non-commercial NuMY assay [37]. It encodes the U2 small nuclear RNA (snRNA). The *RNU2* locus has been classified as a macrosatellite, i.e., a cluster of tandemly arranged repeat units that are longer than those of VNTR loci. Similar to VNTRs, macrosatellites display variability in repeat numbers within the population [46]. The *RNU2* gene cluster is located on chromosome 17 and consists of 20–40 RNU2 repeats, each with a length of 6.1 kb [47,48]. Thus, *RNU2* as a target for DNA quantification has the disadvantage of having far fewer copies than retrotransposons and of inter-individual variations in copy number. Furthermore, the localization at one chromosomal site increases the risk of stochastic sampling effects when analyzing low template DNA (see Figure 2a), and it is not clear how far the degradation of the *RNU2* locus reflects degradation at other chromosomal sites.

Other autosomal non-coding multicopy target loci that have been suggested for forensic DNA quantitation are the 45S ribosomal RNA gene units that are clustered in tandem arrays in about 400 copies on five pairs of autosomes [49]. Each 45S rRNA gene unit contains an array of 5.8S, 18S, and 28S rRNA genes that are separated by spacer sequences and are preceded by a common regulatory sequence. The units are separated by intergenic spacer sequences. While the functionally important rRNA genes are highly conserved across species, their regulatory and intergenic parts are less well conserved and have been used for designing a human-specific qPCR assay called RiboD assay. This assay targets two non-overlapping amplicons of 67 bp and 362 bp in the regulatory region to measure DNA amount and DNA integrity [50]. So far, however, no multiplex assays have been established, and intraindividual and interindividual variations in the sequence and copy number of rRNA genes have been reported [51,52] that might influence quantitation. An advantage of rRNA genes in forensic qPCR would be that their clusters reside on several chromosomes, thus minimizing stochastic sampling effects. Furthermore, the assay principle can readily be transferred to non-human species, thanks to the conservation of rRNA gene arrays across species [50].

### 4.3. Y-Chromosomal Multicopy Loci

Among the protein-coding multicopy genes is Testis-Specific Protein Y-Encoded (*TSPY*), which is located in a tandem array on the short arm of the Y chromosome [53,54,55]. *TSPY* has been shown to be present in 66 copies per genome [53]; however, variability in copy number between human individuals has been reported [54], with low copy numbers correlating with male infertility [56,57]. Due to its Y-chromosomal localization and high copy number, a 133 bp amplicon of the fourth exon of *TSPY* has been suggested for qPCR-based detection of the Y chromosome [58] and as an alternative to amelogenin for forensic sex typing [59]. It is not clear whether this is the same sequence as the 133 bp *TSPY* amplicon targeted by the Plexor HY kit [27].

The non-commercial NuMY assay analyzes a multicopy sequence on the Y chromosome that has been termed YRS but has not been further characterized [60]. A genome search revealed 44 amplicons on the Y chromosome with the predicted lengths of 117 bp to which primers and probes were perfectly matching [37]. The Innoquant HY kit does not disclose the two Y chromosomal multicopy target loci that are analyzed [16]. From both loci, the amplicons have the same size, so the sequence is probably derived from a duplicated region on the Y chromosome. The PowerQuant kit targets two different multicopy loci on the Y chromosome that, however, have not been disclosed [15]. Probably due to the small size difference of the amplicons, they are not suited for specifically analyzing the degradation of the Y chromosome. By contrast, the Investigator Quantiplex Pro kit targets two Y-specific amplicons with considerable length differences (see Table 1) [36] and thus allows for specifically quantitating the degradation of the Y chromosome [34]. The two amplicons reside on the same multicopy locus, which, however, has not been disclosed. The quantitation of the degradation of the Y chromosome may help in predicting Y-STR typing success [34].

### 4.4. Non-Disclosed Target Loci

As mentioned in Section 4.3, for two commercial qPCR assays the Y-chromosomal target loci have not been disclosed. This conforms to a general tendency since for the latest forensic multiplex qPCR kits on the market, none of the target loci are disclosed, and only the amplicon lengths are provided. In the respective validation studies [17,27] and in the technical information provided by the manufacturers, only superficial information is given on the copy numbers and chromosomal localizations (see Table 1). The manual of the Investigator Quantiplex kit names a proprietary sequence (4NS1C) and mentions its copy number and its localization on several autosomes [61]. For both PowerQuant and Quantifiler Trio kits, localization on multiple autosomes and high copy numbers are mentioned in the validation studies, and a generic reference [13] is given for the target loci that, however, does not specify the sequences, copy numbers, or the chromosomal localizations [15,35].

Validation and evaluation studies [14,15,17,27,62] and subsequent successful applications both in experiments and in case work may suffice to trust the qPCR kits in forensic settings and also to reproduce findings obtained using the kits as such. However, the kits then rather resemble black boxes that work “somehow”, and the analyst cannot explain why particular results (e.g., misleading or unexpected results) might have been obtained. For example, it is not clear why the kits display different sensitivities, different levels of accuracy, and different DI values when analyzing different source materials (see Section 5). Moreover, no novel ideas on improvements or on applications in unrelated contexts are stimulated. For example, sequence information might be of interest for considering the usage of the loci in evolutionary studies or for combining the assays with additional amplification targets. As it is feasible to find out the DNA loci by sequencing the amplified products and performing database searches, it is incomprehensible that the identity and nature of the loci have not been revealed by the manufacturers. It appears surprising that initial publications on these kits have been accepted in peer-reviewed journals and that the lack of locus information escaped the reviewers’ attention. In the interest of scientific transparency it is to be hoped that manufacturers will find their way back to scientific standards and will eventually provide this missing information.

## 5. Is There a Relation between the Multicopy Loci and the Performance of a qPCR Assay?

From a theoretical point of view, the chromosomal distributions and copy numbers of target loci would be expected to impact the sensitivity and accuracy of qPCR assays, their performance in quantitating DNA degradation and the male component, and their sensitivity to PCR inhibitors. (The detection of PCR inhibitors is not related to the multicopy targets and has been briefly discussed above; see Section 3.3.) As mentioned, for the latest commercial qPCR assays, the locus information has so far not been disclosed, so any comparisons of their performance must remain descriptive and can at best consider the different lengths of the amplicons used for the quantitation of DNA degradation.

A comparison of the four commercial kits targeting multicopy loci residing on several chromosomes (see Table 1) revealed comparable sensitivities for all of them, with some differences in accuracies at very low DNA concentrations and in precision [14], which might be attributable to stochastic effects due to chromosomal localizations or copy numbers. As summarized in Table 2, the respective developmental validation studies of these kits described their ability to reproducibly quantitate DNA below 1 pg/µL [15,16,17,35], whereas for the Plexor HY kit, DNA concentrations of 1.9 pg/µL were required [27]. The lower sensitivity of the Plexor HY might be related to the clustering of its multicopy target gene at a single chromosomal location, making it more prone to stochastic effects (see Figure 2a).

Because the developmental validation studies determined the sensitivities in slightly different ways, the exact values given are hardly comparable. For example, for the Innoquant HY kit the ability to detect 0.0781 pg/µL DNA for the autosomal targets has been reported [16], which might be attributable to the high copy number of the target loci (see Table 1). However, the sensitivity was not determined according to the Minimum Information for Publication of Quantitative Real-Time PCR Experiments (MIQE) guidelines, which suggest stating the sensitivity as the limit of detection (LOD), i.e., the lowest DNA concentration detectable with 95% certainty [63]. For the Plexor HY, PowerQuant, and Quantifiler Trio kits, the LODs were determined as the lowest concentration yielding quantitation results in all replicates of serially diluted genomic DNA, thus following MIQE guidelines. However, different numbers of replicates and different DNA concentrations were analyzed. For the Investigator Quantiplex Pro kit, it is unclear whether for the reported 0.015625 pg/μL DNA all replicates were detected.

The four commercial qPCR kits indicate degradation by the DI values that are determined with the help of their respective autosomal and degradation targets. For the same degraded DNA sample, the obtained DI values differ between the kits, and consequently, the kits use different DI values to flag samples for moderate or severe DNA degradation (see Table 2). When analyzing degraded DNA, the assay with the longest degradation target (Investigator Quantiplex Pro) showed the highest DI values, whereas the two assays with the smallest degradation targets (Innoquant HY and Quantifiler Trio) showed the smallest DI values [14]. Likewise, another study analyzing degraded DNA from skeletal remains found the lowest DI values for Innoquant HY and Quantifiler Trio [64]. A further study analyzing sheared DNA found higher DI values for the QuantiPlex kit than for the Quantifiler Trio kit [65]. Since the quantitation targets of these kits have roughly similar sizes, these findings are consistent with longer amplicons being more sensitive to degradation. However, despite the almost similar sizes of the degradation and quantitation targets between the kits, a study analyzing DNA from UV-exposed DNA or fingerprints found the Innoquant HY kit to yield higher DI values than the Quantifiler Trio kit [66]. Moreover, in a study by Morrison et al. (2020) comparing the Investigator Quantiplex Pro kit and the PowerQuant kit with sonicated genomic DNA, the former showed higher DI values at DNA concentrations of 250 pg/µL; however, the DI values at the DNA concentration of 25 pg/µL were higher with the latter kit [67]. These two latter studies indicate that for low template DNA, other factors, such as differential sensitivities of the target loci or different copy numbers, might also influence the quantitation of degradation.

Interestingly, in the study by Morrison et al. (2020), the analysis of the degradation of sonicated male genomic DNA with the Investigator Quantiplex Pro kit yielded higher DI values with the Y targets than with the autosomal targets [67]. The almost same sizes of autosomal and Y-chromosomal degradation targets (see Table 1) might suggest a higher sensitivity to DNA degradation of the Y-specific degradation target or of the Y chromosome in general. A recent study by Chierto et al. (2024) seems to point to the same direction. In this study, the Y-specific, longer amplicon of the PlexorHY kit was used as the degradation target to quantitate the autosomal DNA degradation of male DNA samples (with the shorter autosomal amplicon used as the quantitation target), and the results correlated well with the autosomal DNA degradation determined with the PowerQuant kit [68]. Since the size difference of the two amplicons of the PlexorHY kit is much smaller than that of the two autosomal targets of the PowerQuant kit (see Table 1), these findings seem to imply that the TSPY locus might be particularly sensitive to degradation and that Y-specific DNA degradation might serve as an indicator of autosomal DNA degradation in general. It should, however, be noted that the Y-chromosomal targets of the Investigator Quantiplex Pro kit and the PlexorHY kit are present in considerably lower copy numbers than the autosomal targets. Thus, the lower quantification results for the Y targets might simply result from their lower copy numbers because the number of intact copies possibly surviving degradation will be lower.

When the ability to detect the male component in male–female DNA mixtures was compared between the PowerQuant and Investigator QuantiPlex Pro kits, both performed similarly [67]. Another study comparing the detection of the male targets using DNA from skeletal remains showed comparable sensitivities for the Innoquant HY, Quantifiler Trio, and Investigator Quantiplex Pro kits, whereas the PowerQuant kit was less sensitive [64]. As already noted in Section 3.3, the commercial kits differ in their sensitivities to PCR inhibitors [14]. However, as the quantification targets of all kits have roughly the same sizes, their length cannot explain the different sensitivities to PCR inhibition.

Taken together, the latest commercial qPCR kits are generally well suited for quantitating DNA amount and degradation even at low DNA concentrations, as well as for quantitating male components and detecting PCR inhibition. There are, however, subtle differences in performance, and the question remains as to how much these differences are relevant to STR typing success. Several studies have suggested that for optimal STR typing results, the best combination of a qPCR kit and a STR kit should be empirically determined [14,64,65].

## 6. Summary and Outlook

Forensic qPCR assays can help to predict the success of STR analysis and are thus of huge importance in the forensic DNA-analytical work flow. Since forensic qPCR assays specifically quantitate human DNA and use the same method, PCR, that will be used by the subsequent STR analysis, they specifically quantitate the DNA that is actually PCR-amplifiable and thus analyzable. In addition to the quantitation of DNA amounts, forensic qPCR assays provide information on DNA degradation and PCR inhibitors as well and help the analyst decide on the most appropriate analytical steps. The current forensic qPCR assays are adapted to the demands of current downstream STR analysis and are able to analyze DNA concentrations below the sensitivity limits of current STR kits. In keeping pace with further developments of forensic DNA analysis in terms of analytical questions, marker types, sensitivities, robustness, and amplicon lengths, the qPCR assays will likely be further improved in order to carry on giving the most useful information. To foster research on technical improvements and accelerate the emergence of novel ideas, it would be important to make information on the identity of target loci publicly available as per common scientific standards that have ensured scientific progress during the last decades.

For several reasons, in the near future, digital PCR (dPCR) assays will likely be implemented in forensic DNA analysis. As an absolute quantitation method, unlike qPCR, dPCR is not requiring additional quantification standards (for review, see [69]). In dPCR, a defined fraction of the sample is evenly divided into a high number of partitions, such that each contains only a few or no template copies, and PCR-positive partitions are then determined by end-point measurement to calculate back the number of template copies of the original sample. For dPCR, it will be even more important to target multicopy loci that are present on all regions of all chromosomes because it is unpredictable which fraction of a genome (i.e., which chromosomal fragments) will end up in a particular partition. Thanks to end-point measurement, dPCR is only little influenced by variations in PCR efficiencies and is thus less prone to PCR inhibition, but for the same reason, dPCR is also less well suited to detect PCR inhibition [30]. Furthermore, dPCR is able to quantitate minor components in mixtures with a higher sensitivity than qPCR [70]. Since dPCR instruments are able to co-detect several fluorescence colors, and specific PCR products in the partitions can be detected based on fluorescence using TaqMan probes, the design of forensic multiplex dPCR assays is in principle possible [71]. It will be interesting to compare the usefulness of future forensic dPCR assays with qPCR assays for predicting the success of forensic DNA typing.

## Figures and Tables

**Table 1:** Multicopy target loci of current forensic qPCR assays.

Assay (Manufacturer)	Amplicons	DNA Locus	Size bp	Copy Number	Chromosomes	Reference
Innoquant HY (Inno Genomics, New Orleans, LA, USA)	Autosomal long	SVA	207	2762	All	[16]
	Autosomal short	*AluYb8*	80	1733	All	
	Y	Two multicopy targets	79	>3	Y	
Plexor HY ^1^ (Promega, Madison, WI, USA)	Autosomal long	-	-	-	-	[27]
	Autosomal short	*RNU2*	99	20–40	17	
	Y	*TSPY*	133	25–66	Y	
PowerQuant (Promega, Madison, WI, USA)	Autosomal long	ND ^2^	294	High copy number	ND	[15]
	Autosomal short	ND	84	High copy number	ND	
	Y	ND	81 and 136	Two multicopy loci	ND	
Quantifiler Trio Thermo Fisher Scientific (Waltham, MA, USA)	Autosomal long	ND	214	Multicopy	Multiple autosomes	[35]
	Autosomal short	ND	80	Multicopy	Multiple autosomes	
	Y	ND	75	Multicopy	Y	
Investigator Quantiplex Pro (Qiagen, Venlo, The Netherlands)	Autosomal long	4NS1C	353	20	Several autosomes	[36]
	Autosomal short	4NS1C	91	20	Several autosomes	
	Y long	ND	359	Multicopy	Y	
	Y short	ND	81	Multicopy	Y	
NuMY (non-commercial)	Autosomal long	-	-	-	-	[37]
	Autosomal short	*RNU2*	70	20–40	17	
	Y	YRS	117	44	Y	
	mtDNA	mtND1	69	>100	mtDNA	

^1^ Detection principle: Plexor technology; ^2^ ND, not disclosed.

**Table 2:** Sensitivity for autosomal targets and degradation flags of current forensic qPCR assays.

Assay	Long Amplicon	Short Amplicon	Sensitivity	Reference	Degradation Flags ^a^
Innoquant HY	207 bp	80 bp	0.0781 pg/µL	[16]	>10
Plexor HY	-	99 bp	1.9 pg/µL	[27]	-
PowerQuant	294 bp	84 bp	0.5 pg/µL	[15]	>2
Quantifiler Trio	214 bp	80 bp	0.78 pg/µL	[35]	1–10, >10
Investigator Quantiplex Pro	353 bp	91 bp	0.015625 pg/μL	[17]	2.5–20, >20

^a^ Data taken from [14]; for Innoquant HY and PowerQuant, the flags indicating severe degradation are given, and for Quantifiler Trio and Investigator Quantiplex Pro, in addition, the range indicating moderate degradation is given.
